# Thioredoxin Priming Prolongs Lung Allograft Survival by Promoting Immune Tolerance

**DOI:** 10.1371/journal.pone.0124705

**Published:** 2015-05-01

**Authors:** Hanbo Hu, Xiaoyan Zhu, Sunil Joshi, Li Lu, Chang-Qing Xia, Jawaharlal M. Patel

**Affiliations:** 1 Department of Medicine, University of Florida College of Medicine, Gainesville, Florida 32608, United States of America; 2 Department of Pathology, Immunology and Laboratory Medicine, University of Florida College of Medicine, Gainesville, Florida 32608, United States of America; 3 Research Service, North Florida/South Georgia Veterans Health System, Gainesville, Florida 32608, United States of America; Georgia Regents University, UNITED STATES

## Abstract

Tolerance to allograft antigen is the major challenge and final goal of transplant medicine. Our previous study demonstrated that thioredoxin-1 (Trx) priming of donor lung significantly protected allogeneic lung graft. To determine whether Trx priming of donor lung inhibits allograft rejection, extends allograft survival and induces immune tolerance, orthotopic left lung transplantation was performed from Lewis to Sprague-Dawley rats without immunosuppression. Donor lungs were primed with Trx at 4°C for 4 hr prior to transplantation. After up to 37 days post-transplantation, allograft lung morphology, recipient T cell and humoral alloantigen-specific immune responses were examined. We found that Trx-primed lungs exhibited much reduced acute rejection and associated lung injuries resulting in loss of graft functional area at 5-37 days post-transplant in contrast to the control groups. CD4^+^ T cells from the recipients with Trx-primed grafts responded to the stimulation of dendritic cells (DCs) of donor origin, in contrast to DCs from the third party, with significantly reduced proliferation. Consistent with above findings, we observed that CD4^+^Foxp3^+^ regulatory T cells in spleen cells from the recipients with Trx-primed grafts were significantly increased compared to controls, and CD4^+^ T cells from the recipients with Trx-primed grafts produced much higher levels of immunosuppressive cytokine, IL-10 when stimulated with allogeneic donor DCs. In addition, humoral immune tolerance was also induced as there was no significant increase levels of serum antibodies against donor antigens in Trx-lung recipients when re-challenged with allogeneic donor antigens. Our results demonstrate that one-time Trx-priming of donor lung grafts prior to transplantation significantly prolongs the survival of the grafts through inducing or promoting cellular and humoral alloantigen-specific immune tolerance, which might be associated with the induction of immunosuppressive regulatory T cells.

## Introduction

Lung transplantation is the ultimate therapeutic option for end-stage lung diseases. Despite refinement in lung preservation, improvements in surgical techniques, and use of immunosupression regimens, early graft dysfunction and rejection remain a significant cause of morbidity and mortality after lung transplantation. Acute cellular rejection is due to the recipient’s alloreactive T cells which recognize the alloantigen presented in the transplanted tissue and subsequently attack the allograft. Antibody-mediated rejection, on the other hand, is characterized by the development of antibodies to the alloantigen [1]. Although use of immunosuppressive regimens is the current mainstay approach to prevent rejection and promote lung allograft survival, such global immune inhibition leaves the patient susceptible to life-threatening infection, malignancy, organ toxicity, and has limited success in preventing chronic graft rejection [2]. Therefore, selective regulation of the recipient’s immune system to accept the transplanted organ while maintaining normal reactivity against other sources of antigens by alloantigen-specific immune tolerance induction has been the ultimate goal of transplant immunology since the 1950’s [3,4].

We have reported that priming donor lungs with human recombinant thioredoxin-1 (Trx) prior to transplant reduced acute allograft lung injury, transcription factor nuclear factor kappa B (NF-ĸB) activation, and progressive infiltration of inflammatory and cytotoxic CD8^+^ T cells leading to reduced allograft injury without use of immunosuppressant in a rat model of lung transplantation [5]. Trx, a 12-kDa thiol-disulfide oxidoreductase, is a reactive oxygen species (ROS) scavenger and an essential physiological redox regulator of multiple cellular process including gene regulation and cell proliferation involved in inflammatory and immune responses [6–11]. Although a number of chemical and biological agents have been increasingly used to target the immune cascade to modulate immune responses, the specific effects of Trx on immune modulation in lung transplantation remain to be examined. In the present study, we investigated whether one time Trx priming of donor lung prolongs allograft survival and induces immune tolerance.

## Materials and Methods

### Ethics statement

Specific, pathogen-free, male Lewis, Sprague-Dawley (SD), and Wistar rats (300∼350 g) obtained from Harlan (Indianapolis, IN) were used in compliance with the Guide for the Care and Use of Laboratory Animals. The study protocol was approved by the Institutional Animal Care and Use Committee (ACORP number: 0013). All animals were survived during the course of this study. Animals were monitored every 5 to 15 min during recovery period followed by twice a day after recovery for first 48 hr, and daily thereafter for total of 37 days. Any change in bleeding, respiratory distress, body weight, food/water consumption, and signs of infection were monitored for potential post-operative complications. Post-operative analgesic, buprenorphine (0.05 mg/kg, s.c.) was administered twice daily for two days. Post-operative care was monitored and recorded under supervision of facility VMO and his staff. Animals were euthanatized using pentobarbital (60 mg/kg, i.p.) at appropriate experimental end point. All animals were survival to the end point of experiment.

### Surgical Procedures, Treatment, and Assessment of Allograft Acute Rejection

Orthotopic left lung transplants were performed using Lewis donors and SD recipient rats as described [5]. Briefly, donor rats underwent tracheotomy and were mechanically ventilated with 100% O_2_ and 3% isoflurane. Continuous mechanically ventilated with 3.0 cm H2O of positive end-expiratory pressure (PEEP) was performed until the end of flushing. The lungs were flushed through the main pulmonary artery with 20 mL of cold (4°C) Perfadex(Vitrolife, Uppsala, Sweden) with or without Trx (4 μg/ml, USB Corporation, Cleveland, OH) at a pressure of 20 cm H_2_O. When the perfusion is completed the lungs were inflated to 100% of vital capacity and excised. After excision of the heart—lung block, cuffs were attached to the pulmonary artery, pulmonary vein, and bronchus; then, the graft was placed in Perfadex with or without Trx and stored at 4°C for 4 hours. After 4 hours of cold storage, the lung grafts were transplanted into the SD recipients. At sampling, the lung graft was excised and divided into three sections. The upper, middle and lower section of the graft lung, as well as middle section of native lung were embedded in paraffin and used for histological examinations. Hematoxylin and eosin (H&E)–stained lung sections were examined by pathologists to determine the degree of acute cellular rejection (AR) following standardized international grading criteria, 1996 working formulation [12].

### Cell isolation

Recipient’s spleen and allograft lung were removed and CD4^+^ T cells were isolated by positive selection using rat CD4^+^ isolation kit following instructions from the manufacturer (Miltenyi Biotec, Boston, MA). The purity of CD4^+^ T cells was in the range of 90–97%. Naïve Lewis (donor) or Wistar (third party control) splenic dendritic cells (DCs) were isolated and purified by positive selection using OX62^+^ microbeads according to manufacturer’s instructions (Miltenyi Biotec). The purity of OX62^+^ DC cells was 90%.

### Assessment of recipient’s T cell proliferative response *in vitro* and cytokines assay

Recipient spleen CD4^+^ T cells (4 × 10^5^) were cultured with splenic DC (4 × 10^4^) from naïve donor or third party rats for four days in a U-bottom 96-well plate for 5 days. ^3^H-thymidine (1 μCi/well) was added to each culture well for the last 16 h. The cells were harvested and the ^3^H-thymidine incorporation was measured by scintillation counting. Supernatants were pooled from triplicate cultures and cytokine levels were determined using the Milliplex Map Rat Multi-Cytokine Detection Kit for Luminex xMAP technology following the manufacturer’s instructions (Millipore, Billerica, MA).

### Assessment of Foxp3^+^ regulatory T lymphocytes

Recipient’s splenic and allograft lung CD4^+^ T cells were incubated with fluorescein isothiocyanate (FITC)-labeled CD4 antibody followed by intracellular staining for phycoerythrin (PE)-conjugated Foxp3 antibodies according to the manufacturer’s instructions (eBiosciences). The number of CD4^+^Foxp3^+^ T cells was evaluated by FACS-Fortassa flow cytometer and analyzed by FACSDiva (Becton Dickinson, Palo Alto, CA).

### Assessment of *in vivo* humoral tolerance: Measurement of donor antibodies after donor antigen re-challenge to the recipients

Two weeks after transplant, the recipients (SD rats) of lung grafts (from Lewis rats) with or without Trx-priming (n = 3 for each group) were administered by tail vein injection of naïve Lewis spleen cells (SPC, 1 x 10^6^) to re-challenge the recipients once. Serum samples were collected before SPC, one and two weeks after SPC injection. The development of antibodies against donor was determined by immunofluorescent flow cytometry as previously reported [13]. Briefly, naïve donor (Lewis) rat SPC (1.5 x 10^5^) suspended in 20 μl of PBS containing 0.1 percent sodium azide and 1 percent bovine serum albumin (PBS-azide-BSA) were incubated with 10 μl of recipient’s serum at room temperature for 45 minutes. Cells were washed three times and incubated with 30 μl of 40x diluted FITC-labeled goat anti-rat IgG-Fc antibody (Biolegend) for 30 minutes. After three washes, cells were suspended in 0.5 mL of PBS-azide-BSA and the mean fluorescence intensity (MFI) of 1x10^4^ cells was measured in arbitrary units using a FACS-Fortassa flow cytometer and analyzed by FACSDiva (Becton Dickinson, Palo Alto, CA). A pooled, pre-immune rat serum sample was used as a negative control for each experiment.

### Statistical analysis

Experimental results are expressed as mean ± SE. Statistical differences between groups were determined by an unpaired two-tailed Student’s t-test. *P* values less than 0.05 were considered statistically significant.

## Results

### Trx priming attenuates acute allograft rejection and prolongs lung allograft survival

**Fig 1:**
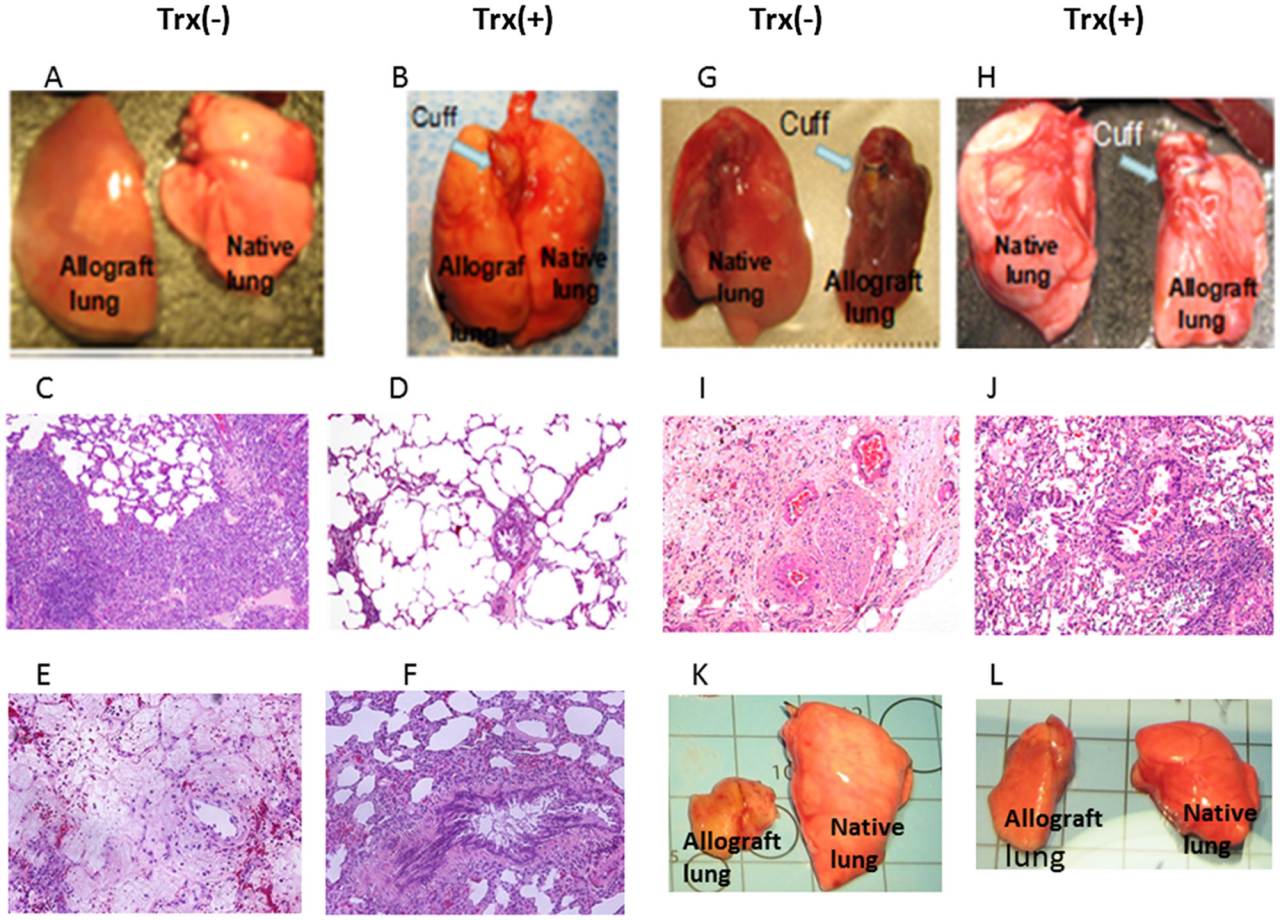
Trx-priming attenuates acute allograft rejection and prolong allograft survival. Orthotopic left lung transplantations from Lewis to Sprague-Dawley rats were performed without immunosuppression using with (+) or without (-) Trx-primed donor lungs. Shown are representative gross images of native lung (right) allograft lung (left) of Trx (-) and Trx (+) priming on day 5 (panels A and B), day 31 (panels G and H), and day 37 (panels K and L) post-transplant, respectively. Shown are representative histological images of Trx (-) and Trx (+) allograft lungs on day 5 (panels C and D), day 7 (panels G and H, and day 31 (panels I and J) post-transplant (original magnification 200x), respectively. Images shown are representative of 3–7 transplants in each group.

We have reported that one time Trx-priming of donor lung significantly attenuate the allograft lung acute injury accompanied with significant increased oxygen exchange function one and five days post transplantation [5]. To determine the effect of Trx on acute allograft rejection and survival, we extended observation to 37 days post transplantation without any immunosuppressant. As shown in Fig 1, control allograft lungs at 5 days post transplantation are enlarged and edematous (panel A). In contrast Trx-primed allograft lungs were near comparable to native lung (panel B). Histological examination shows moderate to severe perivascular and peribronchial mononuclear cell infiltration in control allografts (acute rejection grade A3–A4 and B2–B3) (panel C). Trx-primed allografts show much reduced perivascular and peribronchial mononuclear cell infiltration (acute rejection grade A1–A3 and B1–B2) (panel D) The difference of acute rejection are significant (n = 7 p<0.05 vs control). Allograft lung tissue necrosis occurred at day 7 post transplantation without Trx-priming (panel E), but not in Trx-primed allograft (panel F) (represented one of three). At day 21, 28, 31, and 37 post-transplant (n = 3 in each group), all control allograft lungs shown markedly reduce in size (panels G and K). Histologic examination showed replacement of the lung parenchyma by granulation tissue and fibrosis in control group (panel I) but viable lung with mild to moderate perivascular and peribronchial infiltrate (acute rejection grade A2–3 and B1–B2) in Trx-primed allograft (panel J). At all-time points lung allograft morphology result consistently indicates that Trx-priming of donor lung prolonged allograft survival without use of immunosuppression.

### Trx priming attenuates recipient T cell response to donor alloantigen with increased IL-10 production

To determine whether the improved survival of the Trx-primed allograft is related to T cell tolerance, which is donor-specific low T cell proliferative response to donor alloantigen, purified spleen CD4^+^ T cells from rats transplanted with Trx-priming or control lungs were stimulated with naïve spleen DCs from Lewis donor or third party control Wistar rats in a mixed lymphocyte reaction (MLR). Following DC stimulation, T cell proliferation and IL-10 level in the DC-T cell co-culture media were determined.

**Fig 2:**
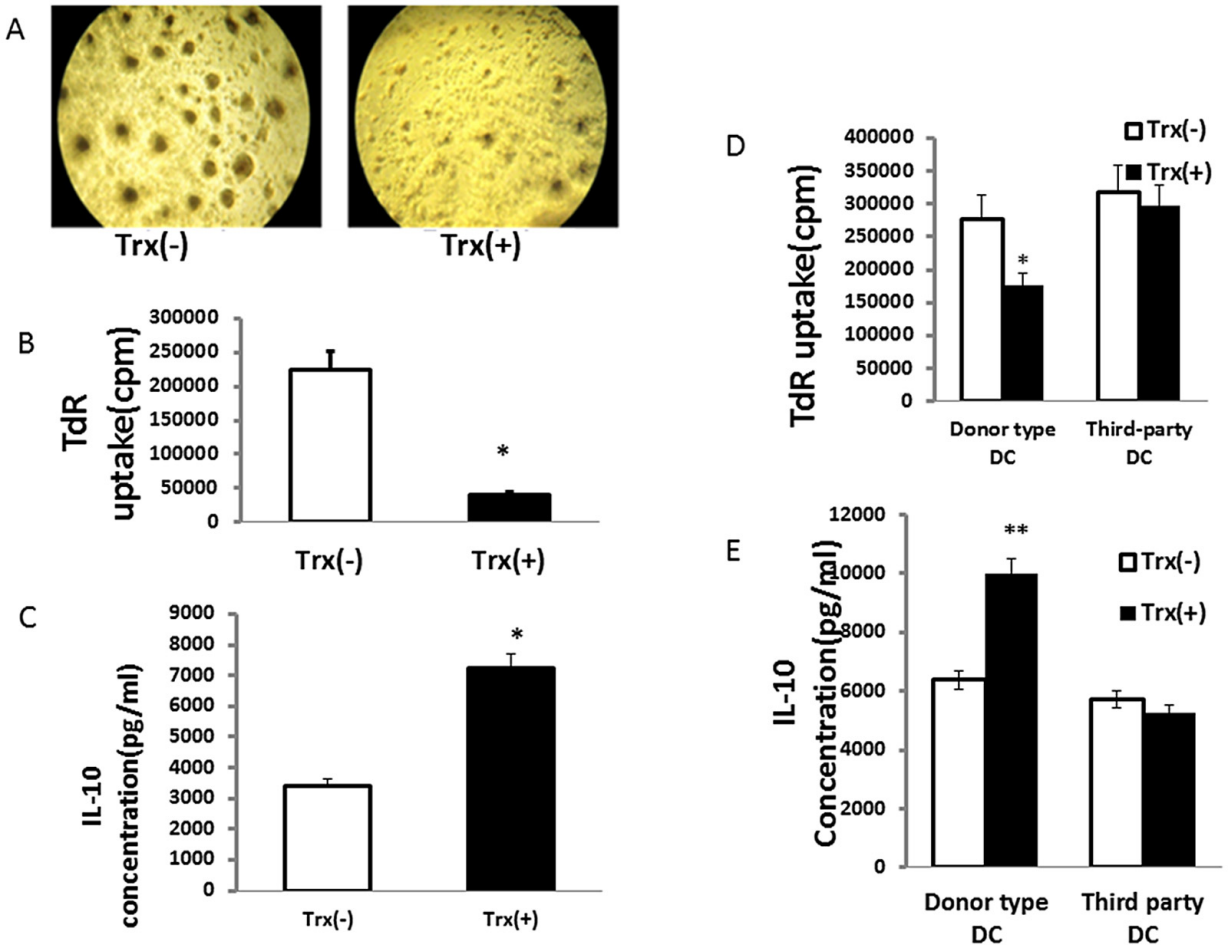
Trx- priming attenuates recipients’ T cell response to the donor type alloantigen accompanied with increased IL-10 production, but not third party control. Orthotopic left lung transplantations were performed from Lewis to Sprague-Dawley rats without immunosuppression using with (+) or without (-) Trx-primed donor lungs. A: Images of T cell proliferating clusters in the co-culture of 7 day transplant recipients’ CD4^+^ T cells and naïve donor Lewis DC at day 3 in MLR, image represents Trx (-) and Trx (+) groups respectively. The round dark areas represent T cell clusters. B: 5 day transplant recipients’ spleen CD4^+^ T cells proliferative response to naive donor DC stimulation in MLR. C: IL-10 concentration in the supernatants of A. D: comparison of 37 day transplanted recipients’ spleen CD4^+^ T cells proliferative response to the stimulation of donor type antigens from Lewis spleen DC and third party antigens from Wistar spleen DC. E: IL-10 concentration in supernatants of D. Data represent mean ± SE, n = 3. (*p<0.05, **p<0.01 vs Trx (-).

This Trx-priming induced inhibition of T cell response was determined at day 5 and 7 post-transplant. Fig 2A shows the images of T cell proliferating clusters in co-culture of host spleen CD4^+^ T cells and donor Lewis DC at 3^rd^ day MLR of Trx(-) (left) and Trx(+)(right) groups. There were fewer and smaller T cell clusters formed in Trx-priming group. This donor DC-stimulated recipient spleen T cell response was accompanied by 80% reduction of T cell proliferation and 113% increase in IL-10 production respectively (Fig 2B and 2C). Similar responses to T cell proliferation and IL-10 production in Trx-primed graft to donor DC stimulation were observed at day 5 post-transplant (data not shown). These results suggested that Trx-priming reduces the host’s T cell response to donor alloantigen after lung transplant with consequent induction of alloantigen-specific IL-10-producing Tr1 regulatory cells.

To further address whether this Trx-inhibited T cell response is donor-specific and whether it can be maintained for an extended period, recipients’ splenic CD4^+^ T cells were isolated 37 days post-transplant and stimulated with naïve DCs from donor or third-party rats. As shown in Fig 2D, Trx-primed donor lung rendered the recipient T cells less proliferative with concomitant increase in IL-10 levels (panel E) in contrast to third party. These results suggest that Trx-priming of allograft induces immune tolerance specifically to donor alloantigen without causing global immunosuppression and this donor-specific tolerance could be maintained for a prolonged period of time in line with 6 weeks post-transplant allograft survival (Fig 1L vs 1K).

### Trx priming enhances recipients’ spleen CD4^+^Foxp3^+^ regulatory T cells

**Fig 3:**
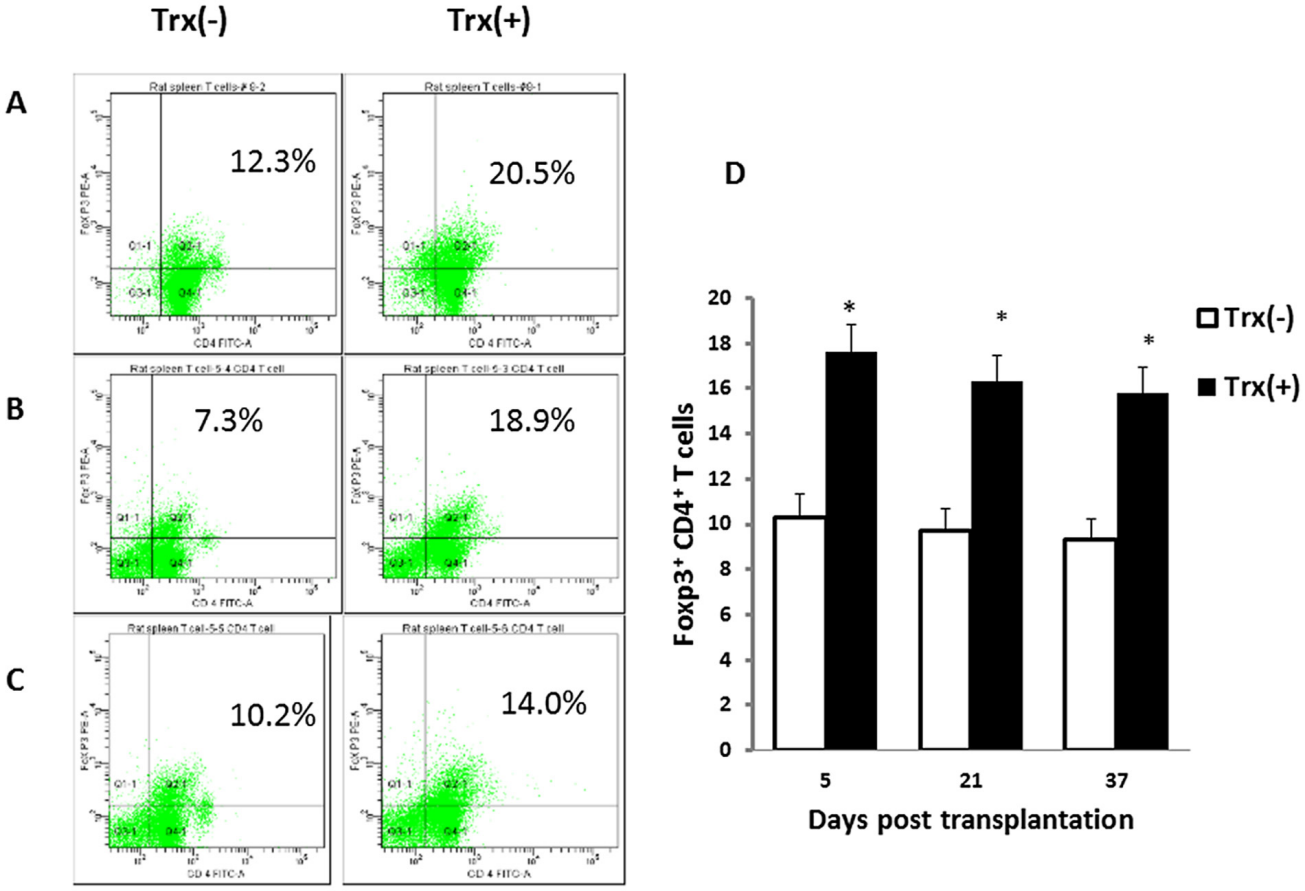
Trx-priming enhances recipients’ spleen CD4^+^Foxp3^+^ regulatory cells. Panels A, B and C shows representative images of one of three separate flow cytometric analysis of spleen CD4 and Foxp3 intracellular staining on day 5, 21 and 37 post-transplant recipients, respectively. Panel D shows quantitatively analysis of host spleen Foxp3^+^ CD4^+^ T cells. Data represent mean ± SE, n = 3.*p<0.05 vs Trx (-).

CD4^+^Foxp3^+^ regulatory T lymphocytes play a critical role in maintaining transplant tolerance in lung transplant patients [14]. In order to determine the underlying mechanism of Trx-priming on suppression of acute allograft lung rejection, we examined the CD4^+^Foxp3^+^ regulatory T cells levels in the recipient’s spleen and 5 day transplanted allograft lung. We found that Trx-priming resulted in significant increase in the levels of recipient’s spleen CD4^+^Foxp3^+^ T cells at days 5, 21 and 37 post lung transplantation compared with control groups (Fig 3). Comparable increase in allograft lung CD4^+^Foxp3^+^ regulatory T cells as in spleen were observed at 5 days post transplantation (data not show). These results indicate that Foxp3^+^ regulatory T lymphocytes may play a critical role in Trx-mediated immune tolerance.

### Trx-priming reduces humoral immune response to donor alloantigen

To determine whether the improved survival of the Trx-primed allograft is also related to humoral tolerance, two weeks post-transplant, recipient rats with or without Trx-primed lungs were given tail vein injection of donor (Lewis rats) spleen cells (SPC) for alloantigen re-challenge once. Serum IgG antibody levels against donor antigens before, 1 and 2 weeks after injection were measured using flow cytometry. The quantitative analysis of serum antibody levels (Panel D) in Pre-SPC Trx (+) group was lower but not significantly different than Pre-SPC Trx (-) group. At 1 and 2 weeks of allogeneic SPC injection, serum IgG levels against donor antigen increased 2–3 fold in Trx (-) group compared to Pre-SPC, however, the serum antibody levels of Pre-SPC, 1 week, and 2 weeks SPC injection in Trx (+) groups remained comparable. The response to the donor SPC re-challenge between Trx (-) and Trx (+) of 1 and 2 weeks were significantly different. These results suggest the induction of humoral immune tolerance to donor antigens by Trx-priming of lung allograft.

## Discussion

**Fig 4:**
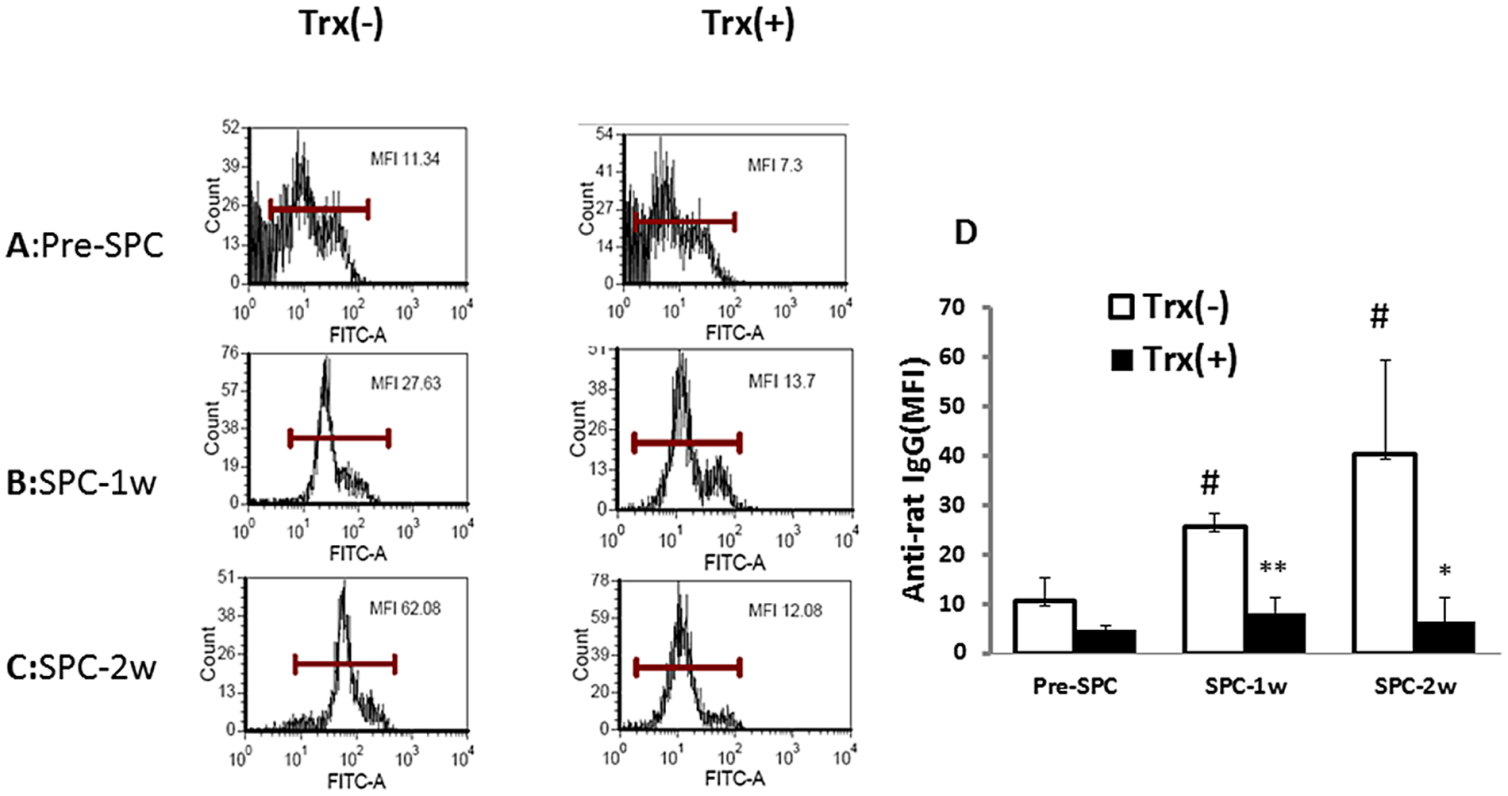
Trx-priming inhibits the production of antibodies against donor type antigen. Two weeks post lung transplant recipient rats were injected with SPC from naïve donor to re-challenge the recipients. The levels of antibodies against donor antigen were measured by immunofluorescent flow cytometry and expressed in MFI. The representative levels of one of three separate host antibodies against donor antigens pre-SPC injection, 1 week, and 2 weeks post-SPC injection are shown in panels A, B, and C, respectively. D: The quantitative levels of host antibodies against donor antigen in pre-SPC, SPC-1 week, and SPC-2 weeks groups (n = 3 for each group). # p< 0.05 vs Trx (-) pre-SPC injection group. *p<0.05, **p<0.01 vs Trx (-) 1 week and 2 weeks, respectively.

The most significant finding of our study is that one time treatment of donor lungs with Trx prior to transplantation reduced acute rejection and induced immune tolerance leading to extended post-transplant allograft survival in a rat model of allogeneic lung transplantation. Trx priming attenuated the recipient’s T cell response to donor alloantigen without compromising other immune functions. It has been reported that in rat allogeneic transplantation, induction of IL-10-producing CD4^+^ Tr1 cells is essential for protecting allograft[15–18]. The increased IL-10-producing CD4^+^ cells in the current study may be involved in the protection of lung graft. CD4^+^Foxp3^+^ regulatory T cells has been shown to play an important role in immune tolerance including allogeneic tolerance [19–23]. Our data showed that CD4^+^Foxp3^+^ regulatory T cells were significantly increased in the Trx(+) lung recipients, suggesting that this treatment induced the development of Foxp3^+^ regulatory T cells, which might contribute to the prolonged graft survival through delivering immunosuppressive signals. Humoral immune tolerance is likely to be induced as well because the anti-donor antibodies remain unchanged after donor antigen challenge in vivo (Fig 4). Antibodies against donor antigens play an important role in chronic allograft rejection [24]. The potential humoral immune tolerance induced could explain the prolonged allograft survival (Fig 2, Fig 4). In addition, we previously reported that the infiltration of CD8^+^ cytotoxic T cells was markedly reduced in the Trx-primed allogeneic lung grafts [5]. Collectively, Trx-priming of lung graft may modulate alloantigen-specific immune responses at different levels. As a result, allograft is protected from immune-mediated destruction.

The precise molecular events associated with Trx priming of donor lung and modulation of the recipient’s immune system leading to extended allograft survival remains to be determined. It is possible that there is a time window, which might be critical for alloantigen-specific immune response to be modulated. Thus, it is speculative in our model that the presence of Trx during the initial contact between recipient immune system and allogeneic lung graft alters the alloantigen-specific immune responses toward protective instead of destructive responses. Increasing evidence supports that reactive oxygen species (ROS) play an essential role in facilitating signal transduction pathway during T cell activation [25,26]. Therefore, Trx-priming may affect alloantigen-specific T cell activation via reduction of ROS by Trx scavenging, or alter T cell differentiation. It has been documented that modulation of ROS production has impact on Th17 cell differentiation [27]. Emerging evidence shows that Th17 plays critical role in lung allograft rejection [28,29]. It is demonstrated that significantly higher immunosuppressive IL-10 is produced by the splenic CD4^+^ T cells from the recipients receiving Trx-primed lung grafts (Fig 2), suggesting the induction of IL-10-producing Tr1 cells. More importantly, those IL-10-producing Tr1 cells are alloantigen-specific, as the stimulation from third party does not lead to increased IL-10 production (Fig 2E). This finding suggests that Trx-priming of lung graft induces alloantigen-specific immune tolerance without affecting immune response globally. Additionally, we found that there were increased levels of Foxp3^+^ CD4^+^ T cells in the recipients receiving Trx-primed allogeneic lung grafts (Fig 3). Those Foxp3^+^ CD4^+^ regulatory T cells have not been fully characterized in terms of their antigen reactivity in this study, but they might have contributed considerably to the immune protection of allogeneic lung grafts. In addition, Trx-priming of lung allograft might also interfere with antigen-presenting cells, such as dendritic cells, thereby affecting subsequent immune responses in which the antigen-presenting cells participate. Indeed, our unpublished data demonstrate that Trx can significantly influence dendritic cells immunophenotypically and functionally.

It is worth noting that in our allogeneic lung transplantation model, the recipients did not receive any immunosuppressive agents, which are routinely employed in the clinical settings of allogeneic lung transplantation [2]. Although the mechanism underlying Trx-priming of allogeneic lung graft induced immune modulation remains to be determined, the translational impact of priming donor lungs with Trx prior to transplantation in a rat model is highly significant in context with its potential utility in clinical settings. Such a strategy offers an excellent non-invasive therapeutic approach to prolong graft survival in lung transplant recipients.
